# Association of peripheral B cells and delirium: combined single-cell sequencing and Mendelian randomization analysis

**DOI:** 10.3389/fneur.2024.1343726

**Published:** 2024-02-06

**Authors:** Siyou Tan, Sining Pan, Lai Wei, Wenyan Chen, Bingbing Pan, Gaoyin Kong, Jing Chen, Yubo Xie

**Affiliations:** ^1^Department of Anesthesiology, The First Affiliated Hospital of Guangxi Medical University, Nanning, China; ^2^Department of Anesthesiology, Hunan Provincial People’s Hospital, The First Affiliated Hospital of Hunan Normal University, Changsha, China; ^3^Guangxi Key Laboratory of Enhanced Recovery After Surgery for Gastrointestinal Cancer, The First Affiliated Hospital of Guangxi Medical University, Nanning, China

**Keywords:** inflammation, delirium, PBMC, B cell, Mendelian randomization

## Abstract

**Background:**

Delirium seriously affects the prognosis of patients and greatly reduces the ability to work and live. Peripheral inflammatory events may contribute to the development of delirium, the mechanism of which is still unclear. There is a lack of effective diagnostic and treatments for delirium in clinical practice. The study aims to investigate alterations in peripheral immune cell subsets under inflammatory stress and to explore causal associations with delirium.

**Methods:**

Single-cell transcriptional sequencing data of human peripheral blood mononuclear cells (PBMC) before and after lipopolysaccharide (LPS) intervention were processed by the Seurat package in R software. PBMC subsets and cellular markers were defined after downscaling and clustering by the Harmony algorithm to identify characteristic subsets in the context of inflammatory stress. Subsequently, a two-sample Mendelian randomization (MR) study was used to explore the causal associations of these inflammation-related PBMC subsets and their molecular phenotypes with delirium. Based on publicly available genetic data, the study incorporated 70 PBMC-associated immune traits, including 8 types of circulating immune cells, 33 B cell subsets and molecular phenotypes, 13 T cell subsets, and 16 B cell-associated cytokines. The results were also validated for robustness, heterogeneity, and horizontal pleiotropy.

**Results:**

Under LPS-induced inflammatory stress, B cells, T cells, monocytes, and dendritic cells in human PBMC showed significant activation and quantitative changes. Of these, only lymphocyte and B cell counts were causally associated with delirium risk. This risk link is also seen in the TNF pathway. Further studies of B cells and their subsets revealed that this association may be related to unswitched memory B cells and CD27 expressed on memory B cells. Annotation of the screened SNPs revealed significant polymorphisms in CD27 and CD40 annotated by rs25680 and rs9883798, respectively. The functions of the key annotated genes may be related to the regulation of immune responses, cell differentiation, proliferation, and intercellular interactions.

**Conclusion:**

The present study revealed the potential possibility that B cell, memory B cell subset, and TNF-related molecules may be involved in the development of delirium due to peripheral inflammation, which can provide clues for further investigation of delirium prevention and treatment strategies.

## 1 Introduction

Delirium is a neurocognitive impairment characterized by attentional deficits, cognitive impairment, and dysfunction of consciousness, which is manifested by a diminished ability to maintain and focus attention, damaged cognition, and disorientation of the environment (1). The prevalence of delirium is significantly higher in older, debilitated populations with comorbid chronic illnesses and those experiencing intense stressful events such as trauma, surgery, and infections (2–4). In contrast to chronic, ongoing neurocognitive impairments (e.g., aging-related and neurodegenerative diseases), the onset of delirium is usually characterized by acute and subacute features (1). For example, aging is often accompanied by cellular senescence and organ dysfunction in the body, accompanied by weakened anti-inflammatory capacity and enhanced pro-inflammatory mechanisms and tends to manifest itself as a natural decline in neurocognitive functioning, which is more physiologic (5). Delirium, on the other hand, often occurs within a short period of time after an intense inflammatory stress event in vulnerable populations, such as delirium occurring for a short period in elderly surgical patients (6, 7). Notably, neurological and cognitive impairments are also frequently observed in chronic diseases such as inflammatory bowel disease, chronic obstructive pulmonary disease, silicosis, coronary heart disease, diabetes mellitus, and nonalcoholic fatty liver disease (8–12), and these populations are at a significantly higher risk of delirium after experiencing intense inflammatory stress (13–15). This phenomenon suggests a possible association of peripheral systems with central neurophysiologic interactions, and suggests that immune and inflammatory alterations in the periphery may induce neurocognitive impairment.

However, there is no clarity regarding the specific mechanisms by which peripheral inflammation leads to delirium. Peripheral effector cells activated during inflammatory stress events as well as transcriptional changes in cytokines and chemokines may be involved in the above process (16). This process may be associated with the induction of neuroinflammation by circulating immune cells and pro-inflammatory mediators through invasion and entry into the damaged blood–brain barrier (BBB) (17). Extensively activated astrocytes and microglia promote inflammatory storm processes in the brain, which ultimately lead to synaptic dysfunction, neuronal loss, and impaired connectivity of functional networks in brain regions (18–20). In patients with neurocognitive impairments, the number and activation status of peripheral blood mononuclear cells (PBMCs) are also often abnormally elevated or decreased, which may be related to the severity and stage of the disease. Different subsets of PBMC may play distinct roles in mediating the interactive effects of the peripheral and central immune environments (21). In the present study, we proposed the hypothesis that PBMC-predominant inflammatory immune activation may have contributed to the development of delirium. Nevertheless, the immune cell subsets and specific effector molecules that specifically mediate peripheral inflammation to the onset of delirium are not known. There are still many gaps in research on delirium causation mechanisms since previous studies have mainly focused on the assessment of learning memory and motor function, but lacked the consideration of delirium characteristics as a whole, which is due to the lack of standardized and feasible methods for assessing delirium-related characteristics (consciousness and attention, etc.), making it difficult to construct an ideal and accepted model of delirium at the experimental animal level (22). Mendelian randomization (MR) studies, on the other hand, have a hierarchy of evidence second only to that of randomized controlled trials, giving the method a strong causal argumentative efficacy. Therefore, we attempted to explore the possible causal associations between various PBMC immune cell subsets and delirium in conjunction with the MR study method after identifying subset alterations of human PBMC under inflammatory stress by analyzing single-cell sequencing data. In this study, we first identified altered characteristic PBMC subsets and their differentially expressed genes by analyzing transcriptome sequencing data of human PBMCs under lipopolysaccharide (LPS)-induced inflammatory stress. Subsequently, single nucleotide polymorphism (SNP) data of relevant PBMC subsets and delirium phenotype were obtained from the human genome-wide association study (GWAS) database, and causal associations between the characterized cell subsets and delirium were explored based on MR methods. This study can screen and identify the characteristic cell types and molecules that may mediate delirium caused by peripheral inflammation, thus providing new ideas and practical references for early diagnosis, prevention, and treatment of delirium.

## 2 Materials and methods

### 2.1 Acquisition of PBMC sequencing data

Human PBMC transcriptome sequencing data were obtained from the GEO database and Human Cell Atlas database to integrate and analyze PBMC subset differences and transcriptional alterations in the inflammatory stress environment. The data were obtained from Human Cell Atlas dataset (dataset 1, https://data.humancellatlas.org/explore/projects/efea6426-510a-4b60-9a19-277e52bfa815) and GSE17842 (dataset 2, https://www.ncbi.nlm.nih.gov/geo/query/acc.cgi?acc=GSE178429). It contains blood samples from 4 and 10 healthy humans, respectively. The samples were categorized into control and LPS groups based on whether or not LPS intervention was performed on the isolated PBMC cells, with 14 samples in each group.

### 2.2 Single-cell RNA sequencing data analysis

De-batching and integration of the two human PBMC transcriptome sequencing datasets and quality control were done by The Seurat package (version 3.0.0) (23, 24). Filtering thresholds were set at (i) cells with less than 200 or more than 2,500 genes, (ii) cells with more than 20% of genes of mitochondrial origin, and (iii) cells with total RNA counts of more than 10,000, as well as genes detected in fewer than 3 cells. After preprocessing, gene expression in the remaining cells was normalized, and subjected to principal component analysis and linear dimensionality reduction to identify significant usable dimensions of the dataset. Subsequently, the Harmony algorithm was used to visualize the clustering classification of all cells (25). Cells with the same features were clustered together after all cells were dimensionally downscaled and projected into two-dimensional space via UMAP. Markers were identified for each clustered cell group using the FindAllMarkers function in Seurat (26). The clusters were then categorized and annotated based on the expression of markers typical of a particular cell type; clusters expressing two or more typical markers were classified as dual cells, while clusters that did not express markers typical of the cell type were classified as low-quality cells. Both dual cell clusters and low-quality cell clusters were excluded from further analysis.

Biological Process (BP) and Kyoto Encyclopedia of Genes and Genomes (KEGG) analyses were conducted on the g: Profiler database,^1^ which analyzes functional enrichment and conversion of gene lists to grasp biological characteristics (27). Differentially expressed genes (DEGs) with *p* < 0.05 as a cut-off criterion were submitted to the g: Profiler database for functional annotation. Targeted drug prediction of druggable genes is accomplished based on the DGIdb database (28). The PPI information was visualized using the Search Tool for the Retrieval of Interacting Genes (STRING) database (29). Then, Cytoscape software was adopted to construct PPI networks (30).

### 2.3 Study design for MR

Based on two-sample MR analyses, we separately assessed causal associations between white blood cells (WBC), lymphocytes (B and T cells), neutrophils, monocytes, and B-cell subsets and delirium. MR uses genetic variation to represent risk factors, so a valid instrumental variable (IV) in causal inference must fulfill three key assumptions: (i) the genetic variation is directly related to the exposure; (ii) the genetic variation is independent of possible confounders between the exposure and the outcome; and (iii) the genetic variation does not affect the outcome through pathways other than the exposure. Ethical approval and informed consent were obtained from participants for all original studies.

### 2.4 Genome-wide association study data sources for MR

Delirium is a severe syndrome of acute or subacute onset of attentional deficits (i.e., diminished ability to point, focus, maintain, and shift attention) and disorders of consciousness (i.e., diminished orientation to the environment) that develops over a short period of time and often fluctuates in symptoms within 1 d and is associated with other cognitive deficits (e.g., memory, language, visuospatial functioning, or perceptual deficits). In the present study, we obtained SNP data for delirium from the FINNGEN database.^2^ The study performed a GWAS on 359,699 European individuals (Number of cases = 3,039, Number of controls = 356,660), with 20 million variants analyzed after quality control and imputation. The dataset was defined as delirium that is not induced by alcohol and other psychoactive substances, including more than 10 types of delirium, such as unspecified delirium, delirium superimposed on dementia, delirium not superimposed on dementia, etc., of which the largest proportion is unspecified delirium, with a major portion of those over 70 years of age (Supplementary Figure S1).

### 2.5 Selection and annotation of instrumental variables

GWAS summary statistics for each immune cell are publicly available from the GWAS catalog (Supplementary Table S1). A total of 70 immune cell-related traits and phenotypes were included, including (i) WBCs, lymphocytes, monocytes, neutrophils, eosinophil, basophil, dendritic cell (DC), and natural killer (NK) cells; (ii) as well as B cell subsets and T cell subsets; and (iii) B cell subsets expressing specific markers (CD20, CD27, CD38, and IgD) and B cell-associated cytokines (IFN-γ, CSF, IL-2, IL-4, IL-6, IL-7, IL-10, and TNF) were also probed for causal delirium associations. There were no overlapping cohorts of European-sourced GWAS data used for the study. We set 1 × e^−5^ as the threshold for screening the significance level of IVs for each immune trait. To remove the chained imbalance status of SNPs, we performed an aggregation process using European reference samples from the 1,000 Genomes Project according to the following thresholds (R^2^ < 0.001, window size = 10,000 kb). Exposure and outcome SNPs were then reconciled to remove ambiguous SNPs that could not be identified as affecting the allele. Palindromic SNPs were specifically examined in the raw dataset to avoid unwanted reverse effects. These rigorously selected SNPs were used as instrumental variables for subsequent two-sample MR analyses. Finally, we used the g: Profiler database for gene annotation and functional enrichment analysis of the screened SNPs.

### 2.6 Statistical analysis

All statistical analyses and graphical representations were done using R version 4.3.2 software and corresponding packages and the GraphPad Prism 9.0. The Seurat package was used for the analysis of PBMC sequencing data (31). The TwoSampleMR package was used for MR analysis (32), which was used to determine the causal relationship between peripheral immunity and delirium. Inverse variance weighting (IVW), weighted median (WM), and MR-Egger methods were mainly used to estimate the causal relationship between exposure and outcome, in which the highest precision and unbiased causal estimates can be provided by IVW (33–36). All GWAS analyses were calibrated using the Bonferroni method. The MR-Egger intercept test was used to assess the effect of horizontal pleiotropy, and Cochrane’s Q test was used to assess the degree of heterogeneity (33, 35). Leave-one-out sensitivity analysis was then used to determine whether the results were affected by any individual SNP, and funnel plots and scatter plots were used to assess heterogeneity. Other software packages used to process the data and generate graphs included the Tidyverse and the Forestplot. Continuous variables are expressed as mean ± SD. Data were tested for normal distribution using the Kolmogorov–Smirnov test. Comparisons between groups of continuous variables were performed using Student’s t test or nonparametric tests. The significance level was set at 0.05 on both sides.

## 3 Results

### 3.1 PBMC subsets alterations under inflammatory stress

**Figure 1:**
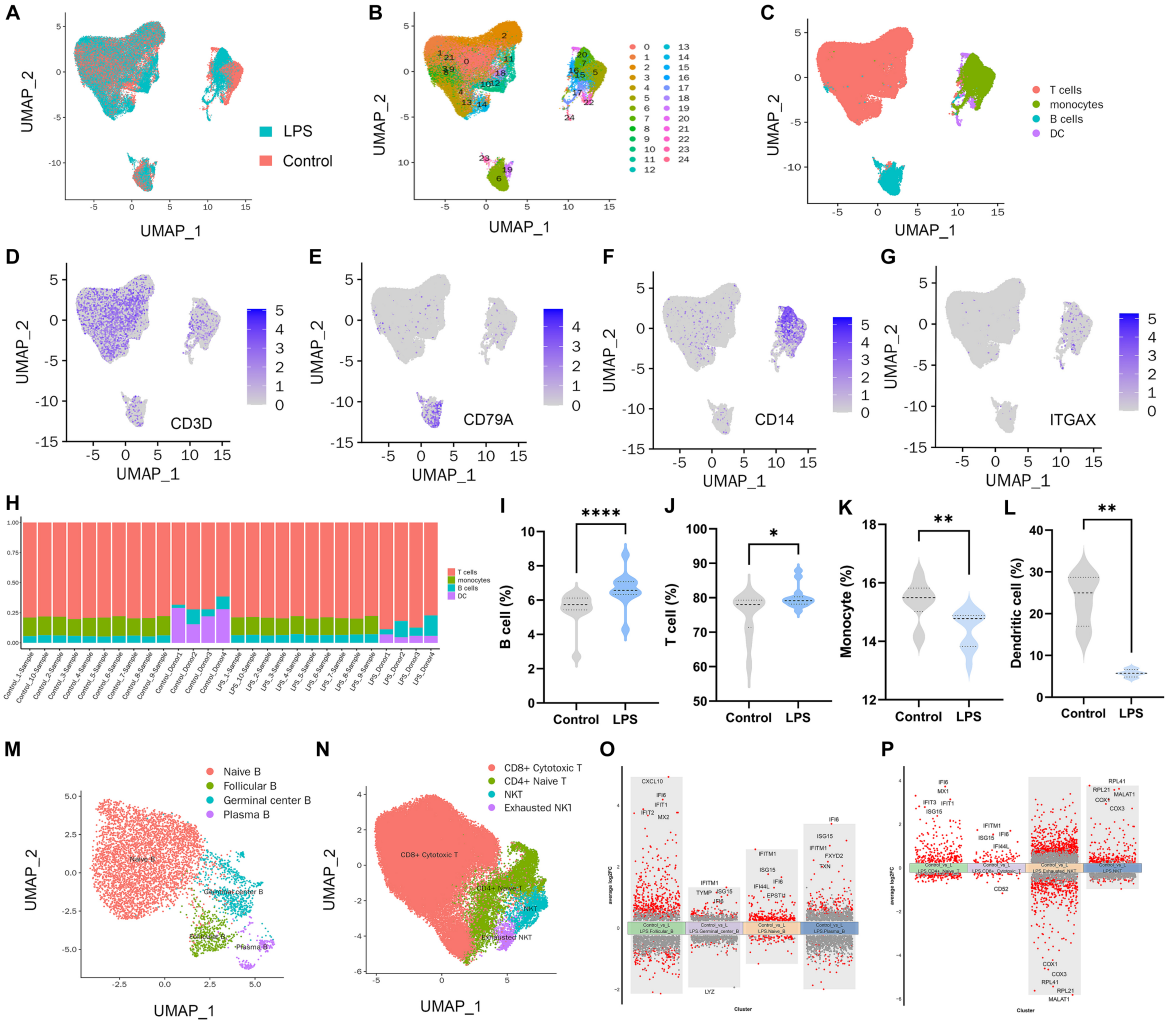
PBMC landscape with and without LPS intervention. **(A)** UMAP plot presenting the control and LPS group in the study. UMAP plot presenting clustering of total PBMCs reveals 24 clusters **(B)** and cell types **(C)**. UMAP plot of CD3D **(D)**, CD14 **(E)**, CD79A **(F),** and ITGAX **(G)** expression on each cell type. **(H)** Percentages of different cell populations in every sample. Percentage of B cell **(I)**, T cell **(J)**, monocyte **(K),** and dendritic cell **(L)** between control and LPS group. **(M)** Annotation of subsets of B cells. **(N)** Annotation of subsets of T cells. **(O)** Volcano maps of differentially expressed genes in B cell subset. **(P)** Volcano maps of differentially expressed genes in T cell subset.

In total, PBMC transcriptome sequencing data were obtained from 14 healthy adults aged 23–37 years before and after the LPS intervention, and there was no difference in the gender composition of the included donors (*p* > 0.05; Supplementary Table S2). After data integration, a total of 50,153 PBMC cells were obtained in the LPS group and 49,276 cells in the control group. After filtering cells with low RNA content or high mitochondrial RNA, we annotated a total of 80,432 cells. By pooling the two data and removing batches (Figure 1A), the unsupervised organization of gene expression revealed 24 clusters and 4 main cell types, including B cells, T cells, monocytes, and DC (Figures 1B,C). Marker genes for each cell type CD3D for T cells, CD79A for B cells, CD14 for monocytes, and ITGAX / CD11c for DCs are shown in Figures 1D–G. Due to missing data for DCs and monocytes in the raw data, which failed to identify DCs or monocytes in Dataset 1 and Dataset 2, respectively, we removed the corresponding samples when counting these two types of cells. In the LPS-induced inflammatory stress environment, T cells were the main PBMC subset affected, followed by DCs, monocytes, and B cells. By comparing the two groups of samples, all four of these cell proportions showed a significant alteration after LPS intervention (Figures 1H–L). Using adjusted *p* value < 0.05 and |log2(fold change)| > 0.5 as the screening threshold, we functionally enriched DEGs in B cells, T cells, monocytes, and DCs, respectively. The results showed that the up-regulated genes in B cells (Supplementary Figure S2A), T cells (Supplementary Figure S2B), DCs (Supplementary Figure S2C), and monocytes (Supplementary Figure S2D) were significantly enriched in immune and inflammatory response activation pathways, and the genes with down-regulated expression were mostly related to bio-metabolic synthesis or cell differentiation. These results suggest that PBMCs exhibit the initiation of immune and defense processes under an LPS-induced inflammatory environment, and lymphocytes and monocytes may be the main effector cells of peripheral inflammatory immune response. Subsequently, we performed further subset annotation of B cells and T cells and found that B cell subsets were mainly clustered into Naïve B cells, follicular B cells, germinal center B cells, and plasma B cells (Figure 1M), while T cells were mainly annotated as CD4+ naïve T cells, CD8+ cytotoxic T cells, NKT cells, and exhausted NKT cells (Figure 1N). We compared the T cell and B cell subsets in the two datasets separately (Supplementary Figure S3). It was found that the proportion of CD4 + naïve T cells, NKT cells, and exhausted NKT cells increased after LPS intervention, while the proportion of naïve B cells decreased, and no statistically significant difference was obtained, although follicular B cells and germinal center B cells showed an upward trend after intervention (Supplementary Figures S3A–C). Based on the conventional understanding, this trend seems reasonable, suggesting an acceleration of T and B cell activation and differentiation processes under inflammatory stress. In particular, naïve B cells undergo accelerated transformation into mature B cells with antibody-secreting activity via the extrafollicular pathway and the germinal center pathway. Identification of DEGs in different B-cell subsets (Figure 1O) and T-cell subsets (Figure 1P) revealed up-regulated interferon-inducible genes such as IFI6, IFITM1, and chemokines such as CXCL10 suggesting a broad spectrum of defense responses and activation of the inflammatory immune response.

### 3.2 Causal effect of genetically predicted PBMC counts, B cell subsets, and T cell subsets on delirium

**Figure 2:**
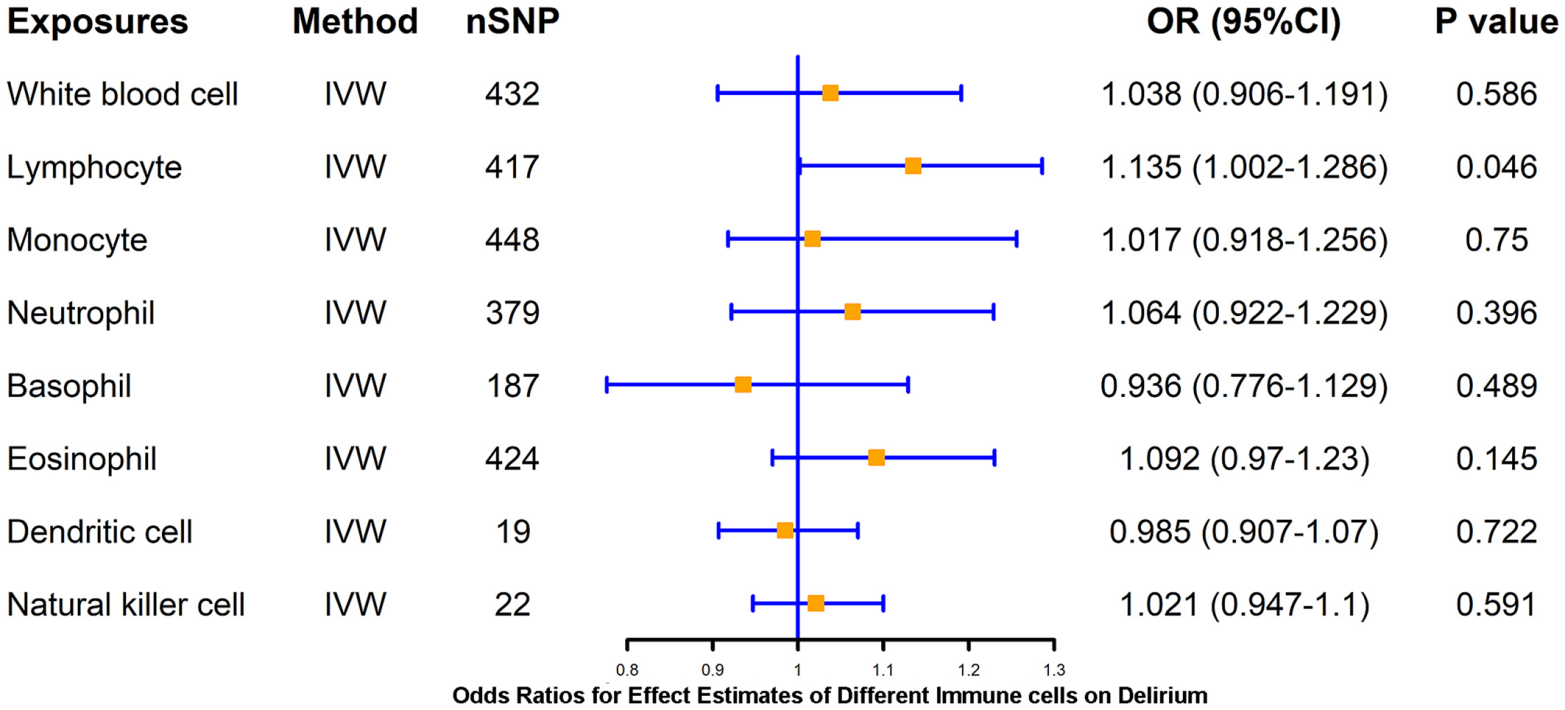
MR estimates of the association between blood cell counts and risk of delirium. IVW, inverse variance weighting; OR, odds ratio; CI, confidence interval.

LPS treatment resulted in significant alterations in the proportion of human-isolated PBMC cells, including B cells, T cells, monocytes, and DCs. Whether activation of these cells mediates delirium due to peripheral inflammation is unclear. To further explore the causal association of different immune cells on delirium, two-sample MR analysis of WBC, lymphocytes, neutrophils, monocytes, eosinophil, basophil, DCs, and NK cell on delirium was performed using the IVW method as the primary method, which resulted in statistically significant causal associations of only lymphocytes (*p* = 0.046) on delirium, suggesting that higher levels of lymphocytes (β = 0.13) may increase the risk of delirium, and WM method also supported this association of lymphocytes with delirium (Figure 2; Supplementary Table S3). We further applied a two-sample MR method to analyze the causal link between different lymphocyte subsets (B cell and T cell) and delirium.

**Figure 3:**
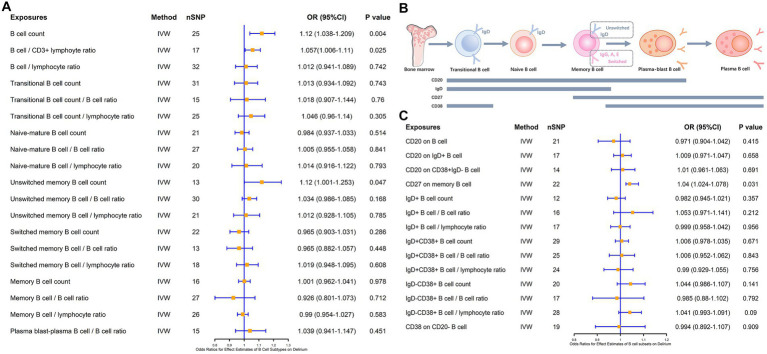
MR estimates of the association between B cell subsets and the risk of delirium. **(A)** MR estimates the association between B cell subsets and the risk of delirium. **(B)** Biomarkers of B cells at different stages of development and maturation. **(C)** MR estimates the association between different B cell markers and the risk of delirium. IVW, inverse variance weighting; OR, odds ratio; CI, confidence interval.

Notably, in addition to Naïve B cells and plasma B cells, follicular B cells and germinal center B cells, which were annotated after the LPS intervention, were associated with both maturation pathways of B cells. Follicular B cells and germinal center B cells can exhibit developmental differentiation during the process of dynamic changes in IgD, CD20, and CD38, so we analyzed the association of various B cell subsets with delirium according to the dynamic changes in cellular markers throughout the course of B cell maturation and differentiation (Figure 3). The different B cells and their subsets included total B cells, transitional B cells, naïve-mature B cells, unswitched memory B cells, switched memory B cells, and plasma B cells and their proportion of lymphocytes. The results revealed that the total B cell count (β = 0.113, *p* = 0.004), the B cell/CD3^+^ lymphocyte ratio (β = 0.056, *p* = 0.025), and unswitched memory B cells (β = 0.114, *p* = 0.047) all have a positive causal link with delirium (Figure 3A). However, none of the B cell subsets such as lgD^+^CD38^+^ B cells, lgD^−^CD38^+^ B cells, and CD20 on IgD^−^ CD38^+^ B cells were causally associated with delirium (Figure 3B). In addition, based on the exploration of markers during B cell subsets development, we found that CD27, as a memory B cell signature marker, was likewise significantly associated with delirium (β = 0.039, *p* = 0.031; Figures 3B,C). Heterogeneity and horizontal multiplicity analyses of the above results demonstrated the robustness of the observed causal associations (Supplementary Table S4). Scatter plots (Supplementary Figure S4), Leave-one-out plots (Supplementary Figure S5), and funnel plots (Supplementary Figure S6) also demonstrated the stability of the results.

**Figure 4:**
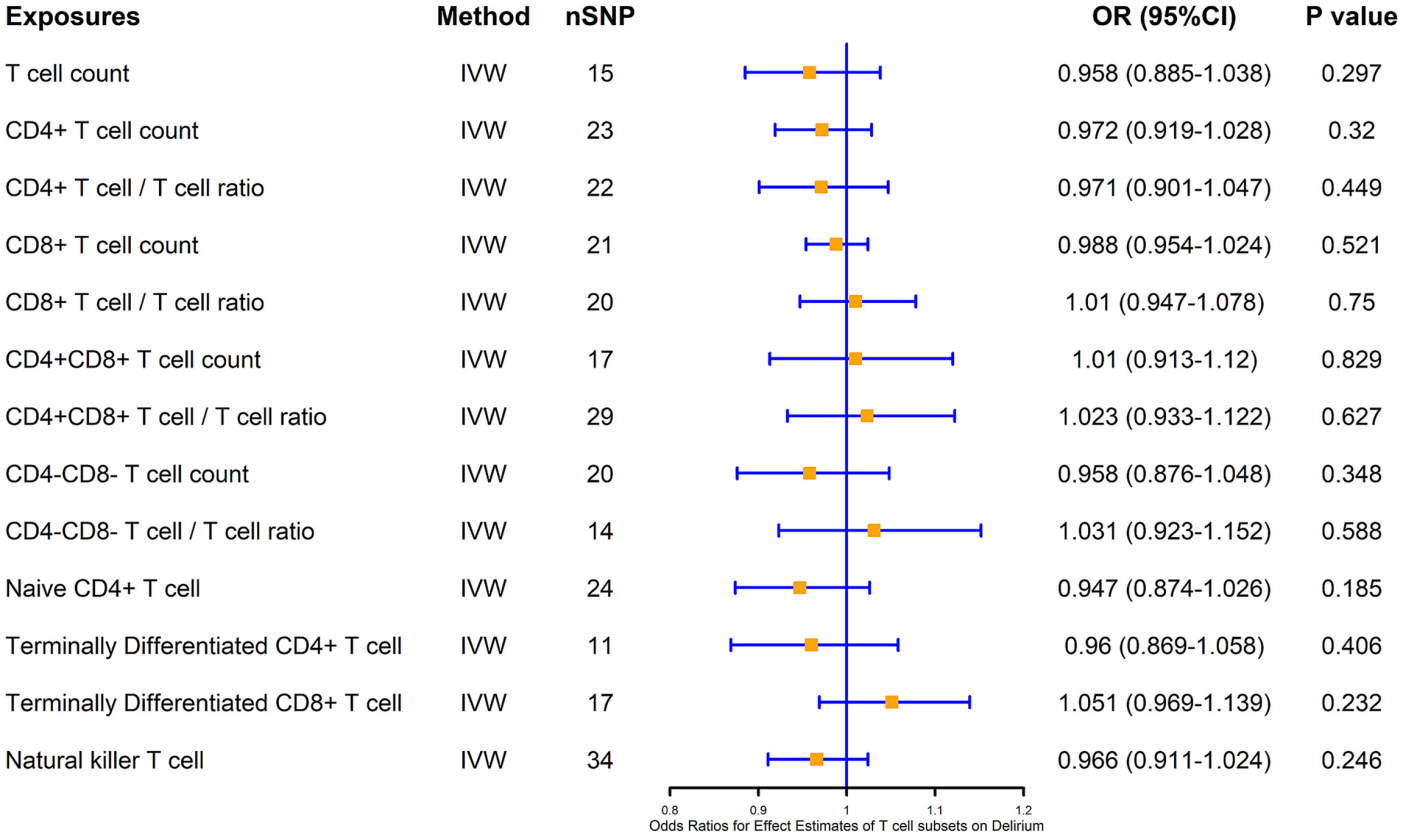
MR estimates of the association between T cell subsets and risk of delirium. IVW, inverse variance weighting; OR, odds ratio; CI, confidence interval.

We also analyzed the causal association of T cells and their subsets with delirium at the same time, and the results showed that there was no significant causal association of CD4^+^ T cells, CD8+ T cells, as well as double-positive (CD4^+^CD8^+^) cells and double-negative (CD4^−^CD8^−^) cells with the risk of delirium causation, and also that there was no significant causal association of naïve CD4^+^ T cells, terminal CD4^+^ T cells, and terminal CD8^+^ T cells with delirium, as well as NKT cells with delirium (Figure 4).

### 3.3 Genetic and functional annotation of potential SNPs

**Figure 5:**
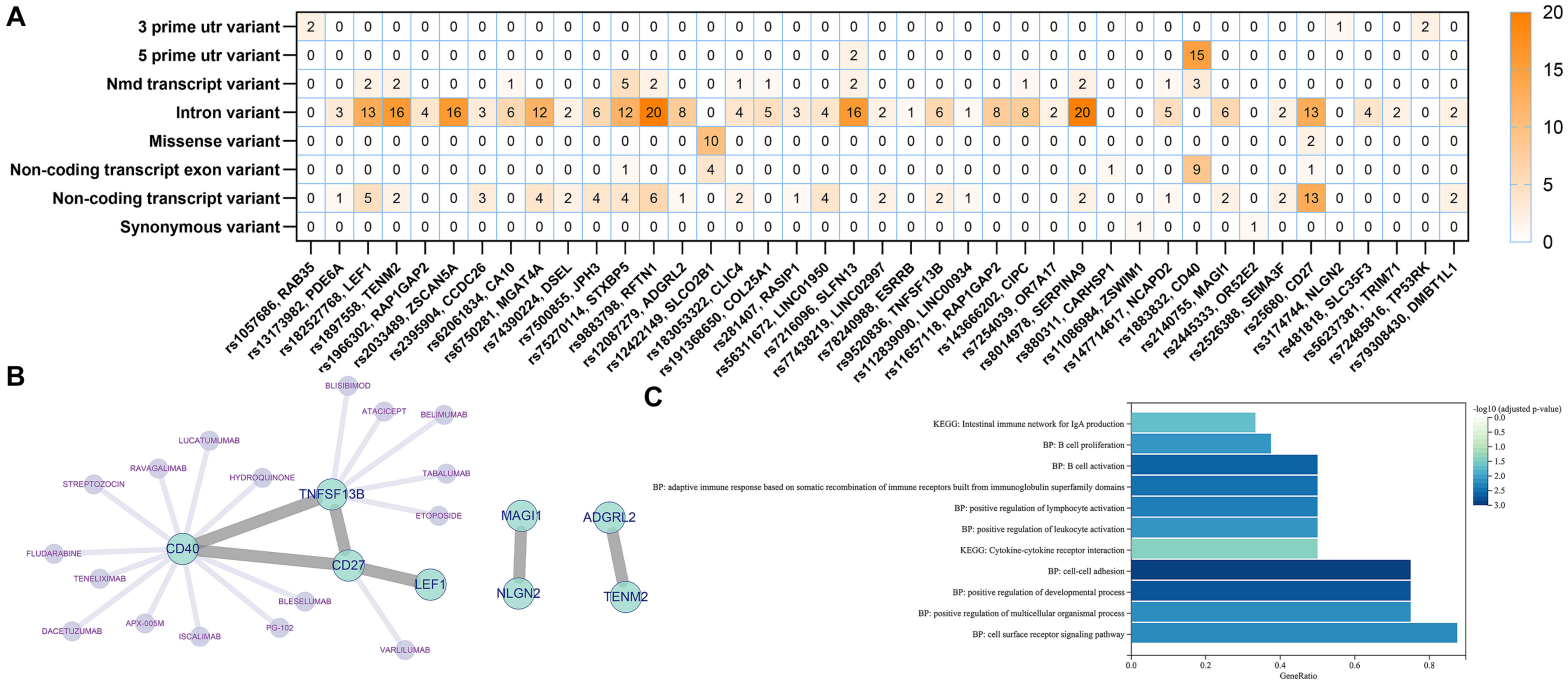
Genetic and functional annotation of potential SNPs. **(A)** Heat map of gene annotation for potential SNPs. **(B)** PPI Networks and targeted drug prediction for annotated Genes. **(C)** Functional enrichment analysis of annotated genes.

We identified a total of 41 genes after annotation of all 76 IVs obtained from all screens in the three exposure events including B cell, unswitched memory B cells, and CD27 on memory B cell. The mutation types and numbers of SNPs corresponding to these 41 genes were plotted in a heat map, the darker the color, the higher the number of mutations that occurred in the SNP site (Figure 5A). The top three SNPs were rs25680, rs9883798, and rs1883832, which were annotated as CD27, RFTN1, and CD40, respectively. Protein–protein interaction (PPI) analysis of the 41 annotated genes revealed that the above three genes together with TNFSF13B constitute the major protein interactions module, suggesting that these SNP loci and genes may contribute a potentially important role in the causal association of B cells with delirium (Figure 5B). The DGIdb database provides predictions for drug targeting of CD27, CD40, and TNFSF3B (Figure 5B). Functional enrichment of the genes in the major module revealed that these genes were significantly associated with the regulation of lymphocyte cell activation, proliferation, and intercellular interactions (Figure 5C).

### 3.4 Causal effect of genetically predicted B cell-associated cytokines on delirium

**Figure 6:**
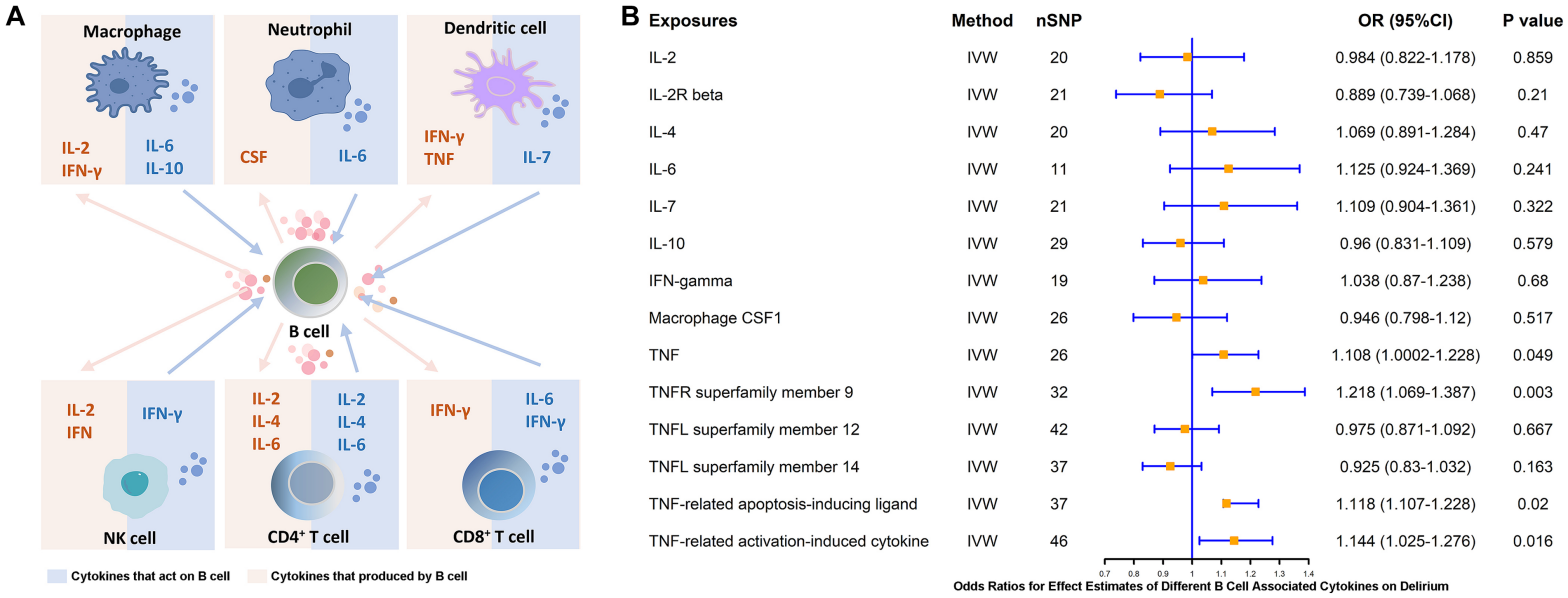
MR estimates of the association between different B cell-associated cytokines and the risk of delirium. **(A)** Cytokines are produced by and acting on B cells. **(B)** MR estimates the association between different B cell-associated cytokines and the risk of delirium. IVW, inverse variance weighting; OR, odds ratio; CI, confidence interval.

B cells, as an essential component of adaptive immunity, possess functions involved in response and cytokine secretion and may exert immunoinflammatory promoting or modulating effects through these associated cytokines. Therefore, we analyzed the major B-cell-associated cytokines, e.g., IFN-γ, CSF, IL-2, IL-4, IL-6, IL-7, IL-10, and key molecules of the TNF pathway, which are involved in B-cell maturation and differentiation as well as in secretory functions (37, 38) (Figure 6A). To further clarify whether these B-related cytokines are associated with the pathogenesis of delirium, we explored the causal link between the two by means of MR studies. The results showed that TNF (β = 0.103, *p* = 0.049) and pathway molecules including TNF receptor superfamily member 9 (CD137 or 4-1BB, β = 0.197, *p* = 0.003), TNF-related apoptosis-inducing ligands (β = 0.111, *p* = 0.02) and levels of TNF-related activation-induced cytokines (β = 0.134, *p* = 0.016) were positively and causally associated with delirium risk (Figure 6B).

## 4 Discussion

Accumulating evidence suggests a correlation between peripheral inflammation and neurocognitive impairment (39, 40). Typical of these, we observed a significantly higher incidence of postoperative delirium in elderly surgical patients, a phenomenon that persists after excluding comorbidities, pain, and anesthesia, suggesting the adverse effects of peripheral stressful events on the central system (3, 41). A systemic homeostatic imbalance characterized by peripheral immune cell activation and a massive increase in inflammation-related mediators may contribute to delirium by impairing the BBB and promoting neuroinflammation formation. Effective identification of the effector cells and molecules that characterize this process could provide avenues for the prevention and treatment of delirium (42, 43).

In the present study, we first identified alterations in cellular subsets of human PBMCs in the presence of inflammatory stress, with B cells, T cells, and monocytes all showing significant increases in response to LPS intervention. We then applied a two-sample MR analysis to explore the correlation between these three cell types and delirium and found that lymphocyte count and B cell count were causally associated with delirium. We then further investigated the causal associations between various subsets of B cells during maturation and delirium and found that unswitched memory B cells and CD27 on memory B cells may be associated with the development of delirium. Based on these results, we further collected SNPs causally associated with delirium, annotated these SNPs, and revealed genes significantly associated with these SNPs. Interaction analysis revealed that CD27 and CD40 were involved in forming an important network of action for these genes, and their associated SNPs showed significant polymorphisms. Functional enrichment of these genes showed a significant correlation with immune cell activation, proliferation, and intercellular interactions. These results suggest that B lymphocytes may be involved in the development of neurocognitive impairment and that this role appears to be related to the complex maturation and developmental stages of B cells. A related study demonstrated that B cells exacerbate neurocognitive impairment in neurodegenerative diseases, showing a significant pro-inflammatory tendency (44). In a recent study by Kim et al. (44), increased B cells in the brains of Alzheimer’s disease (AD) patients, as in the brains of patients with neurocognitive dysfunction due to stroke and multiple sclerosis, exacerbate AD-associated neuroinflammation through the production of immunoglobulins and a range of pro-inflammatory factors. Depletion or inactivation of B cells in the brain increased TGFβ+ microglia and downregulated the expression of TREM2, CLEC7A, and ITGAX in the hippocampus, which significantly slowed the progression of AD (44). It is hypothesized that the neuroinflammation is exacerbated by B cells alone or in synergy with B cell-associated factors. Since B cells are a heterogeneous cell population. Their function and accumulation of subsets are regulated by the inflammatory environment. For example, inflammatory activation ranges from promoting the conversion of innate B1a cells into pathogenic CD137^+^ TNF-α^+^ MHC-Ι^High^ B cells, which then induce cytolytic CD8^+^ T cells and insulin resistance in older adults, rhesus monkeys, and mice (45, 46), the latter of which may promote progression of neurocognitive impairment (47). This suggests that B cells can have an “outside-in” effect on CNS immunopathology (48). In fact, less is known about the involvement of B cells in the onset and progression of neurocognitive impairment. There are differences and discrepancies between different studies and our findings. In human and animal models, higher levels of B cells in the early stages of disease may be beneficial for the progression of neurodegenerative pathology (49, 50). In the early stages of AD, B cell clearance significantly accelerates and exacerbates cognitive deficits and disease progression, leading to increased Aβ burden (50), and depletion of mature B cells similarly exacerbates Aβ load and memory deficits in 5 × FAD mice (49). The differences that contribute to the findings may be related to the complex functional and developmental phenotypes of B cells. As the main effector cells constituting the adaptive immune system, B cells have a variety of cellular and humoral functions that depend on different stages of differentiation and activation. Notably, acute and chronic inflammation have significant differences in pathogenesis, effector immune cells, and inflammation-related molecules (51–53). Thus, the mechanism of B cell effects in chronic neurocognitive impairment disorders may be quite different from that in the acute situation. Currently, there is a paucity of knowledge about the involvement of B cells in the onset and mechanisms of acute neurocognitive impairment.

It has been demonstrated in a large number of studies that proinflammatory cytokines or chemokines are involved in the development of neurocognitive impairment due to peripheral inflammation (16). B cells may exert immunoinflammatory facilitating or modulating roles through these related cytokines. We found that key molecules of TNF and pathways including TNFR superfamily member 9 (CD137 or 4-1BB), TNF-related apoptosis-inducing ligands, and TNF-related activation-induced cytokines had levels were positively and causally associated with delirium risk (Figure 6B). This result likewise supports the previously described pathological process regarding pathogenic CD137^+^ TNF-α^+^ MHC-Ι^High^ B-cell-associated pathology (45, 46). In recent evidence, the median level of serum TNF-α (*p* = 0.048) was significantly higher in patients with delirium after cardiac surgery than in those who did not develop delirium after surgery (54). A trend toward elevated TNF-α can be similarly observed in patients who develop delirium in the ICU (55). In data from a mouse-based study, exposure to elevated TNF-α was found to have strong and acute effects on brain function, including significant activation of neuroinflammation and behavioral changes (56). A systematic review based on 32 studies noted that serum TNF-α and IL-6 are biomarkers of high value for delirium in elderly patients, however, we failed to obtain a potential causal association for IL-6 (57).

We found that among the numerous B cell subsets, both unswitched memory B cells and CD27 on memory B cells were causally associated with delirium. The fate of B cell subsets depends on the expression of specific genes activated and silenced, and therefore their developmental status can be analyzed by the expression of surface and intracellular markers (58). During cell maturation and development, immature B cells from the bone marrow enter the circulatory system and then migrate to the spleen to complete transitional B cell differentiation and eventually become naïve B cells. A small percentage of naïve B cells can differentiate into memory B cells after antigen activation (59). During these processes, IgD expression gradually increases in transitional B cells until it peaks at the naïve B cell stage. IgD and IgM are usually co-expressed on the surface of naïve B cells, and when naïve B cells become memory B cells at an early stage, IgD and IgM are still retained on the cell surface, which is termed as unswitched memory B cells (59). When the IgD and IgM on the surface of unswitched memory B cells are transformed into IgG, IgA, or IgE, they are called switched memory B cells (60). Memory B cells can rapidly recognize and initiate an immune response when stimulated by similar antigens, and reactivate to generate short-lived plasma blasts, which can further mature into plasma B cells (60). B cells at different stages express different surface markers, such as memory B cells characterized by the expression of CD27 and plasma B cells expressing CD38 (60). Although it has been found that no significant differences in overall peripheral B-cell counts have been observed in patients with mild cognitive impairment (MCI) relative to the normal population (61), a growing number of studies have demonstrated potential differences based on B-cell subsets. Poinsatte et al. found that with an increase in peripheral naïve B-cells and a decrease in memory B-cells, patients with MCI showed higher scores on attention and concentration, and executive function index scores were higher (62). Overall cognitive improvement in AD patients was also associated with elevated peripheral naïve B cell levels and decreased memory B cells (62). An overview of the current widespread understanding points to the fact that higher levels of naïve B cells and mature B cells, and fewer memory B cells may be beneficial.

The results of the annotation of the screened SNPs suggest that CD27 and CD40 may be key molecules mediating the involvement of B cells in delirium risk. It is well known that memory B cells often characteristically overexpress CD27, which is important for persistent B cell effector functions and the occurrence of cellular memory (60). CD70, the ligand of CD27, is expressed in mature T cells and B cells. CD27/CD70 induces NF-κB and JNK signaling through the recruitment and activation of TRAF2 and TRAF5, thereby promoting proliferation and differentiation of various types of immune cells and cytokine synthesis (63). CD40 is mainly expressed on antigen-presenting cells (APCs), and CD40/CD40L co-stimulatory signaling promotes APCs functional maturation, which has an important role in thymus-dependent humoral immune responses, regulates CD4^+^ T cells and B cells interactions, and is critical for B cell activation, differentiation, and memory generation (60). There is no shortage of immunotherapeutic strategies developed based on B cells (64). Using the DGIdb database, we predicted potential target drugs for these genes. Among our predicted drugs, as the only predicted CD27 agonistic antibody, Varlilumab has been previously used to explore the treatment of a wide range of hematologic and solid tumors, e.g., in early clinical trials, varlilumab demonstrated preliminary efficacy against hematologic and ovarian cancers (65, 66). CD40 is also expressed in many B-cell tumors and solid tumors. Therefore, CD40 agonistic antibodies such as Dacetuzumab and Lucatumumab also have some anti-tumor potential (67, 68). These drugs achieve therapeutic effects by targeting binding to CD27 or CD40, depleting B cells via antibody-dependent or complement-dependent cell-mediated cytotoxic responses, and direct induction of apoptosis, among other modalities (64). Whether immunotherapeutic strategies targeting B cells may have a positive effect on the prevention and treatment of delirium remains to be further investigated.

Since PBMCs do not include all immune cells in the peripheral circulation, such as polymorphonuclear granulocytes. Among them, neutrophils were shown to play an important role in the inflammatory immune response. Therefore, we analyzed the relationship between various granulocytes and delirium, and although no significant causal associations were obtained, in previous studies neutrophils were shown to possibly mediate central neuroinflammation due to peripheral inflammation, the latter of which further contributes to the decline in learning and cognition. Raymond et al. (18) showed that following distal (caudal) traumatization of the zebrafish resulting in systemic inflammation, leukocytes can invade the brain and increase macrophage, neutrophil, and lymphocyte recruitment and expression of pro-inflammatory mediators in the whole brain/midbrain and forebrain on the first-day post-trauma, inducing an increase in hyperactivity (agitation) and avoidance behaviors. However, there is a lack of clarity regarding the mechanisms by which neutrophils may be involved in the pathogenesis of delirium, mainly owing to the fact that we currently have considerable difficulty in establishing recognized, standardized, and validated animal models of delirium (22, 69). Since delirium is considered to be a syndrome combining impaired consciousness, cognitive impairment, and attentional deficits, there is a lack of validated means of assessing animals on these traits, and common behavioral assessments are aimed at the assessment of learning and memory functions only. Therefore, we can hypothesize that peripheral inflammation and immune activation, such as neutrophil migration and secretion of pro-inflammatory mediators through the damaged BBB promote neuroinflammation synaptic dysfunction, and neuronal loss, which ultimately cause impaired learning and memory function, but do not establish that delirium occurs in the affected animals. On the other hand, the clinical manifestations of delirium may be more complex and severe relative to simple cognitive impairment, and more in-depth studies on the pathogenic mechanisms of delirium are needed.

Our analysis of single-cell sequencing data revealed that both DCs and monocytes showed a decrease in their proportions after LPS intervention. This phenomenon is interesting as it is traditionally recognized that LPS binds to PRRs as an important ligand for receptors on the surface of monocytes or DCs, inducing a pro-inflammatory phenotype. However, this phenomenon is not rare. Measurement of the number of monocytes in the peripheral blood of patients with sepsis has revealed that they exhibit significantly lower numbers compared to normal subjects (70). CD14 expression in 13 human volunteers receiving non-lethal *E. coli* LPS injections revealed that all patients exhibited significant monocytopenia after LPS infusion, with a 52% down-regulation of CD14 expression compared to pre-LPS levels on monocytes obtained 3 h after LPS infusion (70) and that the LPS-induced significant monocytopenia is likewise supported by studies reported by Kathryn et al. (71). In an *in vitro* study, fresh peripheral whole blood was used to assess monocyte and DC responses in co-culture with different doses of LPS. It was found that exposure to all doses of LPS increased monocyte and DC-associated cytokine production and overall leukocyte responses. However, when exposed to higher LPS doses, monocyte and DC cytokine production decreased in a dose-dependent manner. Suggesting a significant effect of LPS concentration on cell subset-specific responses (72). At the same time, we observed a decrease in the proportion of DC after LPS treatment. Alterations in the number and function of DCs in sepsis have been widely reported. Evidence was provided in an earlier study that LPS treatment downregulated the expression of CD11c/ITGAX and CD11b on splenic DC subsets (73). In clinical-based evidence, severe septic shock may lead to a significant decrease in the number of circulating DCs and portends a poor prognosis (74), while sepsis-induced systemic inflammation impairs the ability of hematopoietic stem cells and progenitor cells to produce DCs, including both conventional and plasma cell-like DCs (75). The dramatic decrease in DC numbers in the spleen and peripheral blood during sepsis may be caused by sepsis-associated enhanced apoptosis (76). Several reports have shown that after experiencing intense inflammatory stress, a decrease in DC expression of HLA-DR, CD80, and CD86 can be induced, leading to a decrease in the ability to present antigens (77). In an *in vitro* study, bone marrow-derived DC downregulated CD11c after TLR activation, similar to that after LPS treatment (78). In addition, regarding this interesting phenomenon, we cannot exclude other possible causes, firstly, cell depletion possibly due to sample preservation and handling, because after obtaining peripheral blood samples, one part is used for baseline assays under normal conditions and the other part is used for studies under inflammatory stress, and it is difficult to avoid cell death or depletion due to detachment from physiological environment and sample handling during this process. Secondly, LPS may have induced and accelerated the process of programmed death of monocytes. This phenomenon may be plausible, and the mechanism behind it may be complex, and further studies are needed to help us gain a deeper understanding.

There are some limitations to this study. Due to limited resources in public databases, it was difficult for us to obtain transcriptome sequencing data of PBMC data under LPS intervention for different disease states and different age groups; therefore, targeting healthy adult PBMCs and the corresponding changes induced by LPS may only represent a fraction of the changes that characterize the response of human PBMCs to inflammatory stress, but we hypothesize that such changes are still important, albeit ignoring the age and influences of different disease states. Of interest, delirium itself can occur at any age, from children to older populations. The present study focused on identifying and exploring the subpopulation of PBMC cells in which inflammation may contribute to delirium, and we, therefore, chose a moderately aged, healthy population for the study, which somewhat avoids the interference with cellular stress patterns that may result from different disease states. The triggers of delirium are diverse, such as conditions resulting from severe infections or surgical injuries, and due to limitations in data sources, we were unable to differentiate and investigate the occurrence of delirium for different conditions. In addition, we did not validate the results of this study in patients with clinical delirium. Nor were we able to further assess the efficacy of circulating B cells in the diagnosis of delirium and the significance of guiding clinical outcomes. Further animal experiments are needed to characterize the mechanisms of B cells and their subsets involved in delirium.

## 5 Conclusion

In this study, we combined single-cell transcriptome sequencing data and MR methods to investigate PBMC subsets and their causal association with delirium, revealing the potential association of B cells and memory B cell subset with the occurrence of delirium, which can provide clues for further studies of delirium diagnosis, prevention, and treatment strategies.

## Data availability statement

The original contributions presented in the study are included in the article/Supplementary material, further inquiries can be directed to the corresponding authors.

## Author contributions

ST: Conceptualization, Data curation, Formal analysis, Investigation, Methodology, Software, Visualization, Writing – original draft, Writing – review & editing. SP: Conceptualization, Data curation, Formal analysis, Investigation, Methodology, Software, Visualization, Writing – original draft, Writing – review & editing. LW: Conceptualization, Data curation, Formal analysis, Funding acquisition, Investigation, Methodology, Software, Supervision, Writing – original draft, Writing – review & editing. WC: Conceptualization, Data curation, Formal analysis, Investigation, Methodology, Software, Visualization, Writing – original draft, Writing – review & editing. BP: Conceptualization, Data curation, Formal analysis, Investigation, Methodology, Software, Visualization, Writing – original draft, Writing – review & editing. GK: Conceptualization, Data curation, Formal analysis, Investigation, Methodology, Software, Visualization, Writing – original draft, Writing – review & editing. JC: Conceptualization, Data curation, Formal analysis, Investigation, Methodology, Software, Supervision, Visualization, Writing – original draft, Writing – review & editing. YX: Data curation, Formal analysis, Funding acquisition, Investigation, Methodology, Software, Supervision, Visualization, Writing – original draft, Writing – review & editing, Conceptualization.

## Footnotes

^1^ https://biit.cs.ut.ee/gprofiler/gost

^2^ https://www.finngen.fi/en
