# Supplementary material for: Association of peripheral B cells and delirium: combined single-cell sequencing and Mendelian randomization analysis
Source: Front Neurol. 2024 Feb 6;15:1343726. doi: 10.3389/fneur.2024.1343726 (PMC10876872; doi:10.3389/fneur.2024.1343726)
Supplement: Supplementary file 1 [file Table_1.DOCX]

**Table S1 Description of included traits in the study.**

| **Traits** | **Sample size** | **Number of SNPs** | **Author** | **Population** | **GWAS ID** |  |
| --- | --- | --- | --- | --- | --- | --- |
| **Exposure** |  |  |  |  |  |  |
| White blood cell | 563,946 | - | Vuckovic, et al. | European | ieu-b-30 |  |
| Lymphocyte | 563,946 | - | Vuckovic, et al. | European | ieu-b-32 |  |
| Neutrophil | 563,946 | - | Vuckovic, et al. | European | ieu-b-34 |  |
| Monocyte | 563,946 | - | Vuckovic, et al. | European | ieu-b-31 |  |
| Eosinophil | 563,946 | - | Vuckovic, et al. | European | Ieu-b-33 |  |
| Basophil | 563,946 | - | Vuckovic, et al. | European | ieu-b-29 |  |
| Dendritic cell | | 3,374 | 18,332,528 | Valeria Orrù, et al. | European | GCST90001461 |
| Natural killer cell | | 3,653 | 15,195,758 | Valeria Orrù, et al. | European | GCST90001645 |
| B cell | | 3,653 | 15,195,758 | Valeria Orrù, et al. | European | GCST90001642 |
| B cell / CD3+ lymphocyte ratio | 3,669 | 15,198,020 | Valeria Orrù, et al. | European | GCST90001643 |  |
| B cell / lymphocyte ratio | 3,669 | 15,198,020 | Valeria Orrù, et al. | European | GCST90001644 |  |
| Transitional B cell count | 3,656 | 15,048,937 | Valeria Orrù, et al. | European | GCST90001577 |  |
| Transitional B cell count / B cell ratio | 3,657 | 15,048,951 | Valeria Orrù, et al. | European | GCST90001576 |  |
| Transitional B cell count / lymphocyte ratio | 3,656 | 15,048,937 | Valeria Orrù, et al. | European | GCST90001578 |  |
| Naive-mature B cell count | 3,656 | 15,048,937 | Valeria Orrù, et al. | European | GCST90001409 |  |
| Naive-mature B cell / B cell ratio | 3,657 | 15,048,951 | Valeria Orrù, et al. | European | GCST90001408 |  |
| Naive-mature B cell / lymphocyte ratio | 3,656 | 15,048,937 | Valeria Orrù, et al. | European | GCST90001437 |  |
| Unswitched memory B cell count | 3,656 | 15,048,937 | Valeria Orrù, et al. | European | GCST90001398 |  |
| Unswitched memory B cell / B cell ratio | 3,657 | 15,048,951 | Valeria Orrù, et al. | European | GCST90001397 |  |
| Unswitched memory B cell / lymphocyte ratio | 3,656 | 15,048,937 | Valeria Orrù, et al. | European | GCST90001432 |  |
| Switched memory B cell count | 3,656 | 15,048,937 | Valeria Orrù, et al. | European | GCST90001403 |  |
| Switched memory B cell / B cell ratio | 3,657 | 15,048,951 | Valeria Orrù, et al. | European | GCST90001402 |  |
| Switched memory B cell / lymphocyte ratio | 3,656 | 15,048,937 | Valeria Orrù, et al. | European | GCST90001434 |  |
| Memory B cell count | 3,656 | 15,048,937 | Valeria Orrù, et al. | European | GCST90001407 |  |
| Memory B cell / B cell ratio | 3,657 | 15,048,951 | Valeria Orrù, et al. | European | GCST90001406 |  |
| Memory B cell / lymphocyte ratio | 3,656 | 15,048,937 | Valeria Orrù, et al. | European | GCST90001436 |  |
| Plasma blast-plasma B cell / B cell ratio | 3,658 | 15,049,172 | Valeria Orrù, et al. | European | GCST90001404 |  |
| CD20 on B cell | 3,660 | 15,049,199 | Valeria Orrù, et al. | European | GCST90001743 |  |
| CD20 on IgD+ B cell | 3,658 | 15,048,965 | Valeria Orrù, et al. | European | GCST90001762 |  |
| CD20 on CD38+IgD- B cell | 3,659 | 15,049,184 | Valeria Orrù, et al. | European | GCST90001756 |  |
| CD27 on memory B cell | 3,657 | 15,048,951 | Valeria Orrù, et al. | European | GCST90001805 |  |
| IgD+ B cell count | 3,657 | 15,048,951 | Valeria Orrù, et al. | European | GCST90001400 |  |
| IgD+ B cell / B cell ratio | 3,658 | 15,048,965 | Valeria Orrù, et al. | European | GCST90001391 |  |
| IgD+ B cell / lymphocyte ratio | 3,657 | 15,048,951 | Valeria Orrù, et al. | European | GCST90001424 |  |
| IgD+CD38+ B cell count | 3,656 | 15,048,937 | Valeria Orrù, et al. | European | GCST90001392 |  |
| IgD+CD38+ B cell / B cell ratio | 3,657 | 15,048,951 | Valeria Orrù, et al. | European | GCST90001447 |  |
| IgD+CD38+ B cell / lymphocyte ratio | 3,656 | 15,048,937 | Valeria Orrù, et al. | European | GCST90001429 |  |
| IgD-CD38+ B cell count | 3,656 | 15,048,937 | Valeria Orrù, et al. | European | GCST90001420 |  |
| IgD-CD38+ B cell / B cell ratio | 3,657 | 15,048,951 | Valeria Orrù, et al. | European | GCST90001410 |  |
| IgD-CD38+ B cell / lymphocyte ratio | 3,656 | 15,048,937 | Valeria Orrù, et al. | European | GCST90001425 |  |
| CD38 on CD20- B cell | 3,630 | 15,042,909 | Valeria Orrù, et al. | European | GCST90001809 |  |
| T cell | | 3,653 | 15,195,758 | Valeria Orrù, et al. | European | GCST90001603 |
| CD4+ T cell | | 3,652 | 15,195,743 | Valeria Orrù, et al. | European | GCST90001590 |
| CD4+ T cell / T cell | | 3,668 | 15,198,002 | Valeria Orrù, et al. | European | GCST90001591 |
| CD8+ T cell | | 3,652 | 15,195,743 | Valeria Orrù, et al. | European | GCST90001592 |
| CD8+ T cell / T cell | | 3,668 | 15,198,002 | Valeria Orrù, et al. | European | GCST90001593 |
| CD4+CD8+ T cell | | 3,652 | 15,195,743 | Valeria Orrù, et al. | European | GCST90001594 |
| CD4+CD8+ T cell / T cell | | 3,668 | 5,946,402 | Valeria Orrù, et al. | European | GCST90001595 |
| CD4-CD8- T cell | | 3,652 | 15,195,743 | Valeria Orrù, et al. | European | GCST90001598 |
| CD4-CD8- T cell / T cell | | 3,668 | 15,198,002 | Valeria Orrù, et al. | European | GCST90001599 |
| Naïve CD4+ T cell | | 3,395 | 15,129,224 | Valeria Orrù, et al. | European | GCST90001540 |
| Terminally Differentiated CD4+ T cell | | 3,395 | 15,129,224 | Valeria Orrù, et al. | European | GCST90001545 |
| Terminally Differentiated CD8+ T cell | | 3,395 | 15,129,224 | Valeria Orrù, et al. | European | GCST90001557 |
| Natural killer T cell | | 3,653 | 15,195,758 | Valeria Orrù, et al. | European | GCST90001621 |
| IL-2 | | 1,585 | 12,685,297 | Zhao JH, et al. | European | GCST90274806 |
| IL-2 receptor subunit β | | 1,585 | 12,686,983 | Zhao JH, et al. | European | GCST90274811 |
| IL-4 | | 1,585 | 12,687,001 | Zhao JH, et al. | European | GCST90274813 |
| IL-6 | | 8,189 | 10,700,000 | Ahola-Olli AV, et al. | European | GCST004446 |
| IL-7 | | 1,585 | 12,958,029 | Zhao JH, et al. | European | GCST90274816 |
| IL-10 | | 1,585 | 12,958,183 | Zhao JH, et al. | European | GCST90274795 |
| IL-10 receptor subunit α | | 1,585 | 12,687,028 | Zhao JH, et al. | European | GCST90274796 |
| IL-10 receptor subunit β | | 1,585 | 12,957,474 | Zhao JH, et al. | European | GCST90274797 |
| IFN-γ | | 1,585 | 12,687,006 | Zhao JH, et al. | European | GCST90274794 |
| Macrophage colony-stimulating factor 1 | | 1,585 | 12,957,362 | Zhao JH, et al. | European | GCST90274776 |
| TNF | | 5,358 | 7,506,463 | Gudjonsson A, et al. | European | GCST90089150 |
| TNFR superfamily member 9 | | 1,585 | 12,686,010 | Zhao JH, et al. | European | GCST90274841 |
| TNFL superfamily member 12 | | 1,585 | 12,958,021 | Zhao JH, et al. | European | GCST90274846 |
| TNFL superfamily member 14 | | 1,585 | 12,687,022 | Zhao JH, et al. | European | GCST90274842 |
| TNF-related activation-induced cytokines | | 1,585 | 12,958,022 | Zhao JH, et al. | European | GCST90274844 |
| TNF-related apoptosis-inducing ligands | | 9,173 | 20,300,000 | Folkersen L, et al. | European | GCST90012011 |
| **Outcome** |  |  |  |  |  |  |
| Delirium | 359699 | - | - | European | F5_DELIRIUM |  |

SNP, single nucleotide polymorphism; GWAS, genome-wide association study; IFN, interferon; IL, interleukin; TNF, tumor necrosis factor; TNFL, tumor necrosis factor ligand; TNFR, tumor necrosis factor receptor.
