# Supplementary material for: Association of peripheral B cells and delirium: combined single-cell sequencing and Mendelian randomization analysis
Source: Front Neurol. 2024 Feb 6;15:1343726. doi: 10.3389/fneur.2024.1343726 (PMC10876872; doi:10.3389/fneur.2024.1343726)
Supplement: Supplementary file 2 [file Table_2.DOCX]

**Table S2 Basic characteristics of PBMC donors.**

|  | **Sample for control （number）** | **Sample for LPS （number）** | **Sex (M/F)** | **Age (year)** |
| --- | --- | --- | --- | --- |
| **PBMC Donor 1** | 1 | 1 | F | 27 |
| **PBMC Donor 2** | 1 | 1 | M | 31 |
| **PBMC Donor 3** | 1 | 1 | M | 27 |
| **PBMC Donor 4** | 1 | 1 | F | 29 |
| **PBMC Donor 5** | 1 | 1 | M | 24 |
| **PBMC Donor 6** | 1 | 1 | F | 23 |
| **PBMC Donor 7** | 1 | 1 | F | 32 |
| **PBMC Donor 8** | 1 | 1 | M | 27 |
| **PBMC Donor 9** | 1 | 1 | M | 27 |
| **PBMC Donor 10** | 1 | 1 | F | 25 |
| **PBMC Donor 11** | 1 | 1 | F | 24 |
| **PBMC Donor 12** | 1 | 1 | M | 25 |
| **PBMC Donor 13** | 1 | 1 | M | 26 |
| **PBMC Donor 14** | 1 | 1 | M | 37 |

PBMC, peripheral blood mononuclear cell.
