# Supplementary material for: Association of peripheral B cells and delirium: combined single-cell sequencing and Mendelian randomization analysis
Source: Front Neurol. 2024 Feb 6;15:1343726. doi: 10.3389/fneur.2024.1343726 (PMC10876872; doi:10.3389/fneur.2024.1343726)
Supplement: Supplementary file 3 [file Table_3.DOCX]

**Table S3 MR estimates of the association between immune traits and risk of delirium.**

| **Trait** | **Method** | **nSNP** | **β** | **SE** | **P-value** | **OR** | **95% CI _Low_** | **95% CI _Up_** |
| --- | --- | --- | --- | --- | --- | --- | --- | --- |
| **Lymphocyte** | IVW | 417 | 0.127 | 0.064 | 0.046 | 1.135 | 1.002 | 1.286 |
|  | MR Egger | 417 | 0.215 | 0.137 | 0.118 | 1.239 | 0.948 | 1.621 |
|  | WM | 417 | 0.268 | 0.108 | 0.013 | 1.307 | 1.058 | 1.615 |
| **B cell** | IVW | 25 | 0.113 | 0.039 | 0.004 | 1.12 | 1.038 | 1.209 |
|  | MR Egger | 25 | 0.061 | 0.067 | 0.374 | 1.063 | 0.932 | 1.212 |
|  | WM | 25 | 0.075 | 0.056 | 0.179 | 1.078 | 0.966 | 1.203 |
| **B cell / CD3^+^ lymphocyte ratio** | IVW | 17 | 0.056 | 0.025 | 0.025 | 1.057 | 1.006 | 1.11 |
|  | MR Egger | 17 | 0.044 | 0.031 | 0.183 | 1.045 | 0.982 | 1.111 |
|  | WM | 17 | 0.045 | 0.032 | 0.157 | 1.046 | 0.983 | 1.113 |
| **Unswitched memory B cell count** | IVW | 13 | 0.114 | 0.057 | 0.047 | 1.12 | 1.001 | 1.253 |
|  | MR Egger | 13 | 0.18 | 0.147 | 0.247 | 1.197 | 0.897 | 1.596 |
|  | WM | 13 | 0.155 | 0.079 | 0.049 | 1.168 | 1.001 | 1.363 |
| **CD27 on memory B cell** | IVW | 22 | 0.039 | 0.018 | 0.031 | 1.04 | 1.024 | 1.078 |
|  | MR Egger | 22 | 0.027 | 0.023 | 0.253 | 1.027 | 0.982 | 1.074 |
|  | WM | 22 | 0.032 | 0.023 | 0.154 | 1.033 | 0.988 | 1.079 |
| **TNF** | IVW | 26 | 0.103 | 0.0523 | 0.049 | 1.109 | 1 | 1.228 |
|  | MR Egger | 26 | 0.183 | 0.127 | 0.162 | 1.201 | 0.936 | 1.541 |
|  | WM | 26 | 0.045 | 0.073 | 0.535 | 1.046 | 0.907 | 1.208 |
| **TNFR superfamily member 9** | IVW | 32 | 0.197 | 0.067 | 0.003 | 1.218 | 1.069 | 1.387 |
|  | MR Egger | 32 | 0.41 | 0.146 | 0.009 | 1.506 | 1.131 | 2.006 |
|  | WM | 32 | 0.153 | 0.097 | 0.114 | 1.166 | 0.964 | 1.41 |
| **TNF-related apoptosis-inducing ligands** | IVW | 37 | 0.111 | 0.048 | 0.02 | 1.118 | 1.017 | 1.228 |
|  | MR Egger | 37 | 0.124 | 0.073 | 0.101 | 1.132 | 0.98 | 1.307 |
|  | WM | 37 | 0.085 | 0.068 | 0.211 | 1.089 | 0.953 | 1.244 |
| **TNF-related activation-induced cytokines** | IVW | 46 | 0.134 | 0.056 | 0.016 | 1.144 | 1.025 | 1.276 |
|  | MR Egger | 46 | 0.055 | 0.111 | 0.626 | 1.056 | 0.849 | 1.313 |
|  | WM | 46 | 0.066 | 0.077 | 0.389 | 1.069 | 0.919 | 1.243 |

MR, Mendelian randomization; OR, odds ratio; CI, confidence interval; SNP, single nucleotide polymorphism; SE, standard error; IVW, inverse variance weighted; WM, weighted median; TNF, tumor necrosis factor; TNFL, tumor necrosis factor ligand; TNFR, tumor necrosis factor receptor.
