# Supplementary material for: Association of peripheral B cells and delirium: combined single-cell sequencing and Mendelian randomization analysis
Source: Front Neurol. 2024 Feb 6;15:1343726. doi: 10.3389/fneur.2024.1343726 (PMC10876872; doi:10.3389/fneur.2024.1343726)
Supplement: Supplementary file 4 [file Table_4.DOCX]

**Table S4 Results of heterogeneity and horizontal pleiotropy.**

|  | **Heterogeneity** | |  | **Pleiotropy (Egger intercept test)** | |
| --- | --- | --- | --- | --- | --- |
|  | **MR-IVW (P-value)** | **MR-Egger (P-value)** |  | **Statistics** | **P-value** |
| **Lymphocyte** | 0.553 | 0.561 |  | 0.01 | 0.365 |
| **B cell** | 0.75 | 0.752 |  | 0.012 | 0.346 |
| **B cell/CD3^+^ lymphocyte ratio** | 0.267 | 0.237 |  | 0.011 | 0.528 |
| **Unswitched memory B cell** | 0.697 | 0.752 |  | -0.013 | 0.636 |
| **CD27 on memory B cell** | 0.628 | 0.634 |  | -0.002 | 0.47 |
| **TNF** | 0.474 | 0.444 |  | -0.011 | 0.492 |
| **TNFR superfamily member 9** | 0.573 | 0.661 |  | -0.025 | 0.113 |
| **TNF-related apoptosis-inducing ligands** | 0.901 | 0.879 |  | -0.002 | 0.825 |
| **TNF-related activation-induced cytokines** | 0.181 | 0.174 |  | 0.01 | 0.41 |

MR, Mendelian randomization; IVW, inverse variance weighted; TNF, tumor necrosis factor; TNFL, tumor necrosis factor ligand; TNFR, tumor necrosis factor receptor.
