# Supplementary material for: Association of peripheral B cells and delirium: combined single-cell sequencing and Mendelian randomization analysis
Source: Front Neurol. 2024 Feb 6;15:1343726. doi: 10.3389/fneur.2024.1343726 (PMC10876872; doi:10.3389/fneur.2024.1343726)
Supplement: Supplementary file 5 [file Data_Sheet_1.docx]

**Figure S1**

**
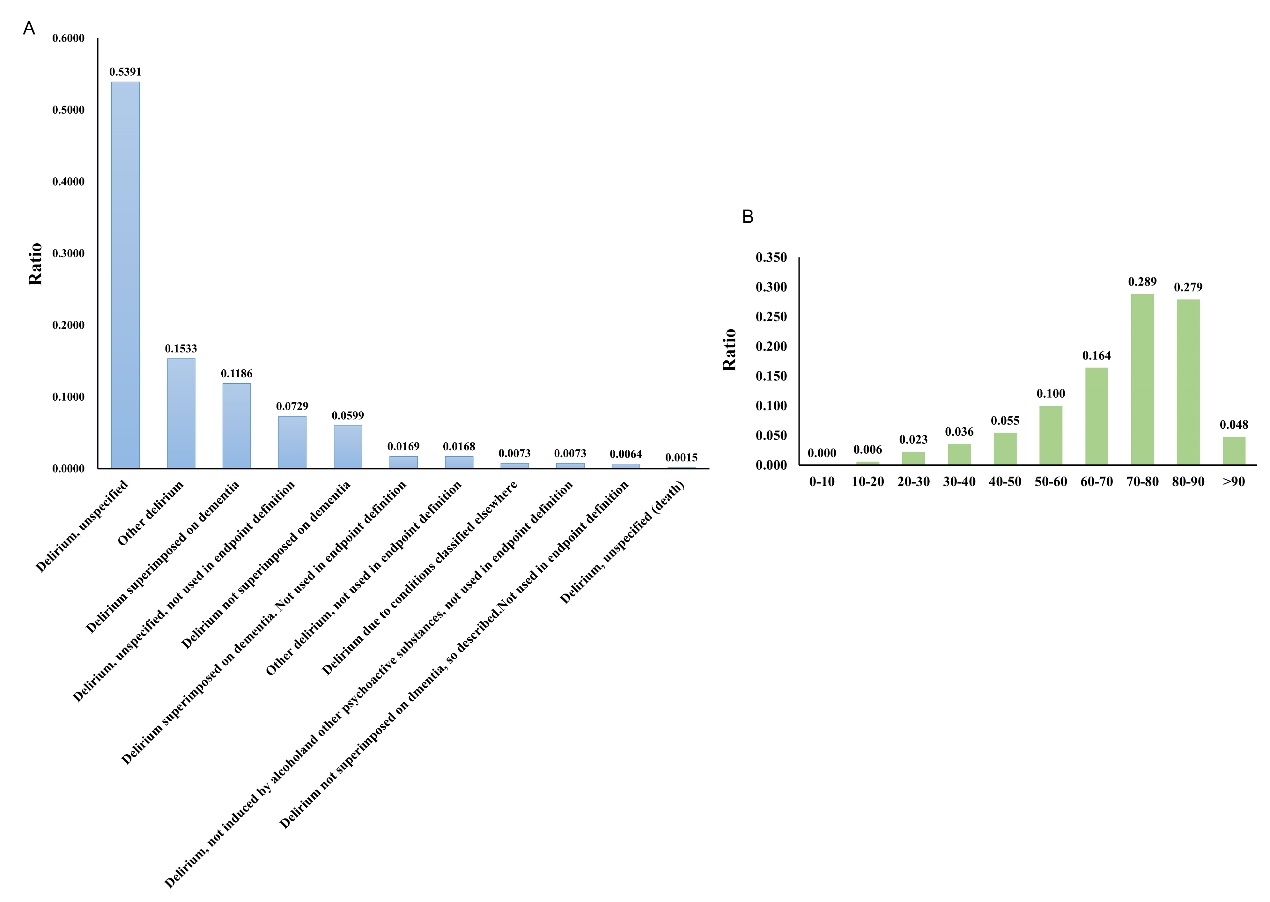
**

**Figure S1 Types and age composition of delirium outcome.** (A) The proportion of delirium types; (B) Age (year) composition of delirium cases.

**Figure S2**

**
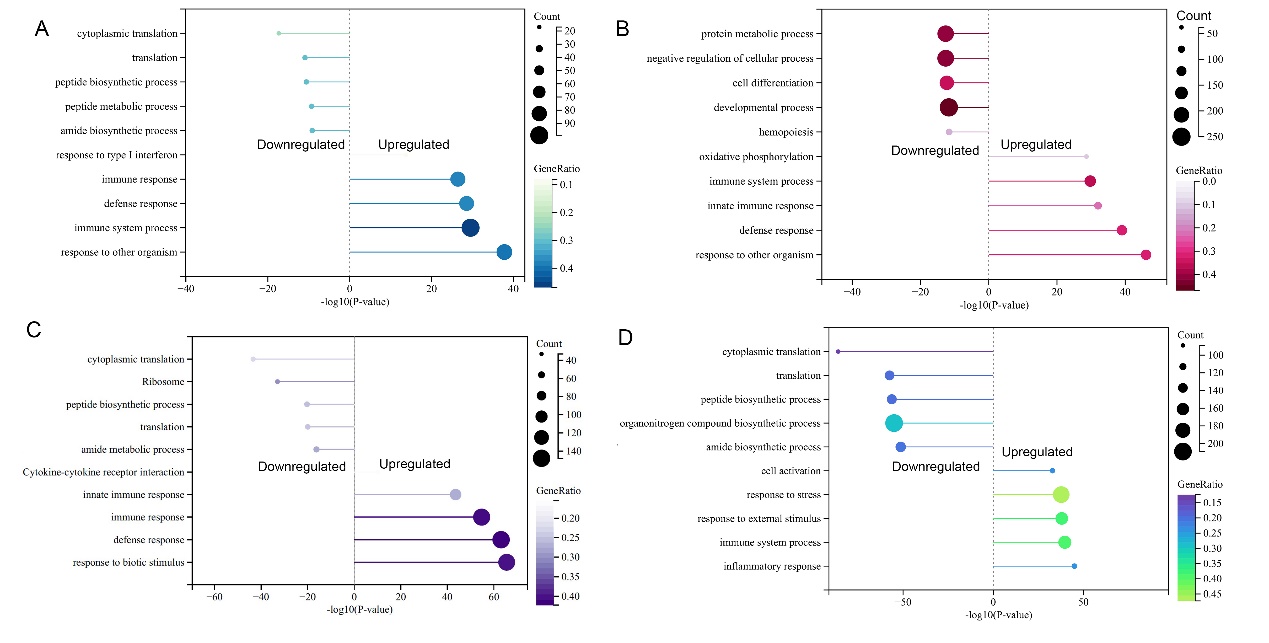
**

**Figure S2 Functional enrichment analysis.** Functional enrichment analysis of DEGs in B cell (A), T cell (B), dendritic cell (C), and monocyte (D) cluster.

**Figure S3**

**
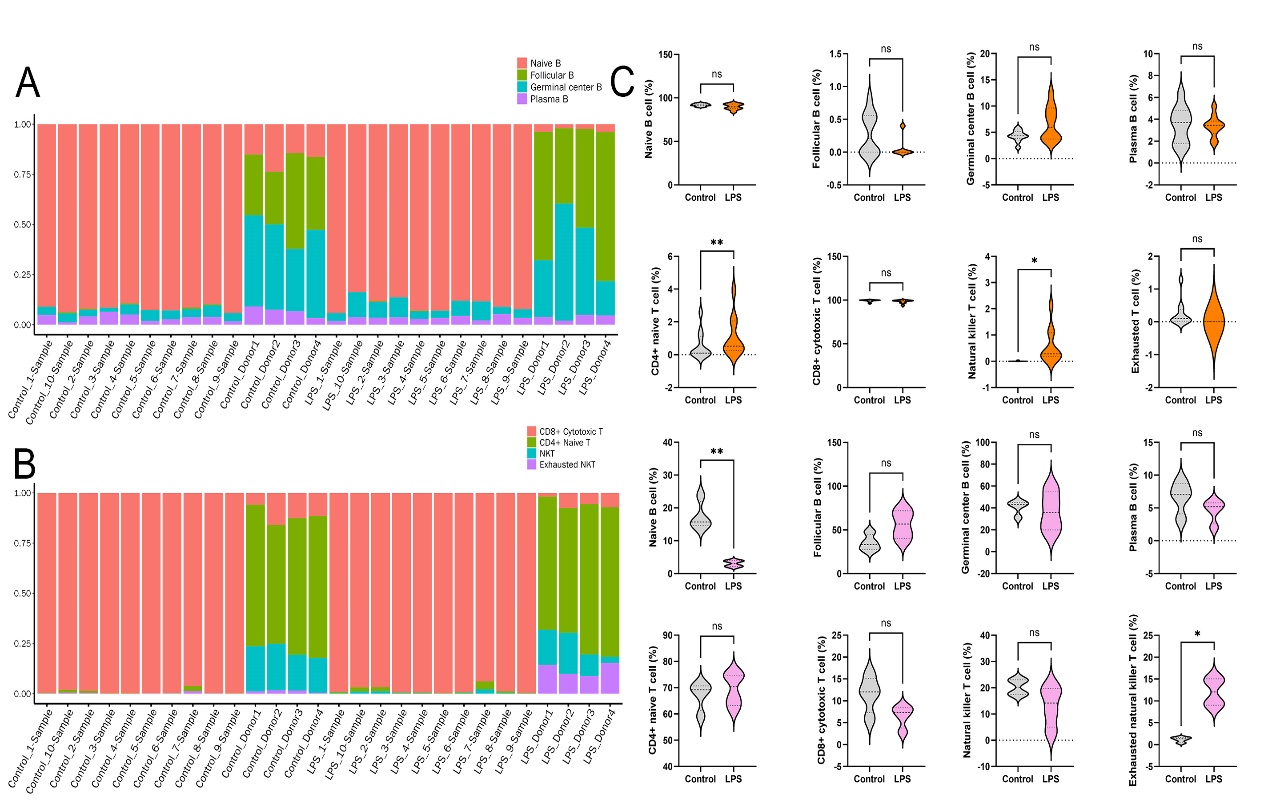
**

**Figure S3 Proportion of B-cell and T-cell subsets in each sample. (A)** Proportion of B-cell subsets in each sample. **(B)** Proportion of T-cell subsets in each sample. **(C)** Comparison of the proportions of each cell subset between the two groups (orange, n=10, dataset 1; pink, n=4, dataset 2).

**Figure S4**


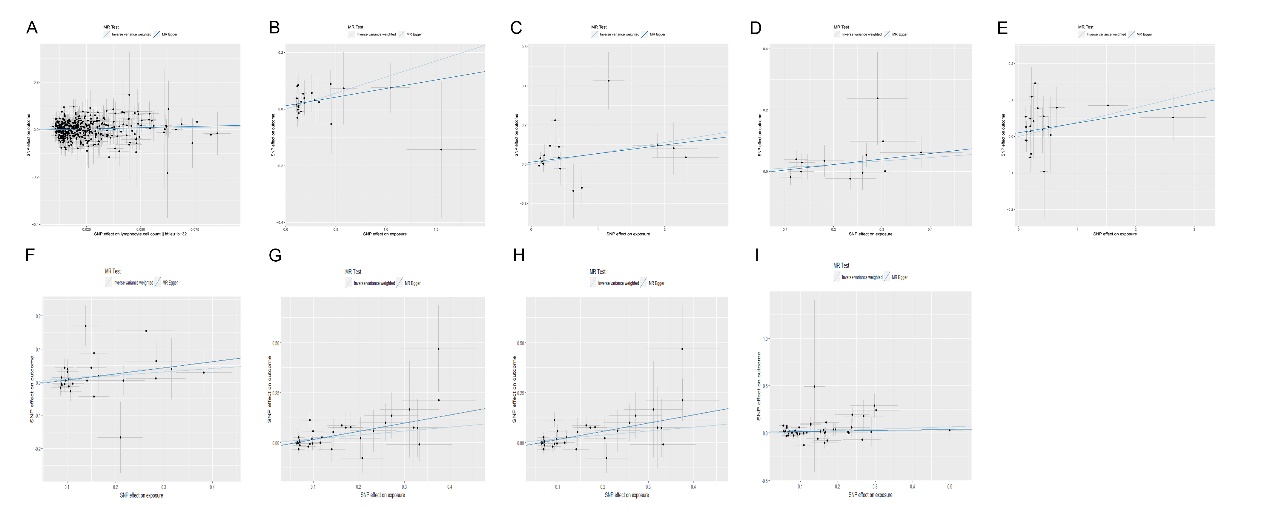


**Figure S4 Scatter plots of MR results for nine immune traits.** Scatter plots of MR results for lymphocyte **(A)**, B cell **(B)**, B cell / CD3+ lymphocyte ratio **(C)**, unswitched memory B cell **(D)**, CD27 on memory B cell **(E)**, TNF **(F)**, TNFR superfamily member 9 **(G)**, TNF-related apoptosis-inducing ligands **(H)**, TNF-related activation-induced cytokines **(I)**. MR, Mendelian randomization; TNF, tumor necrosis factor; TNFL, tumor necrosis factor ligand; TNFR, tumor necrosis factor receptor.

**Figure S5**

**
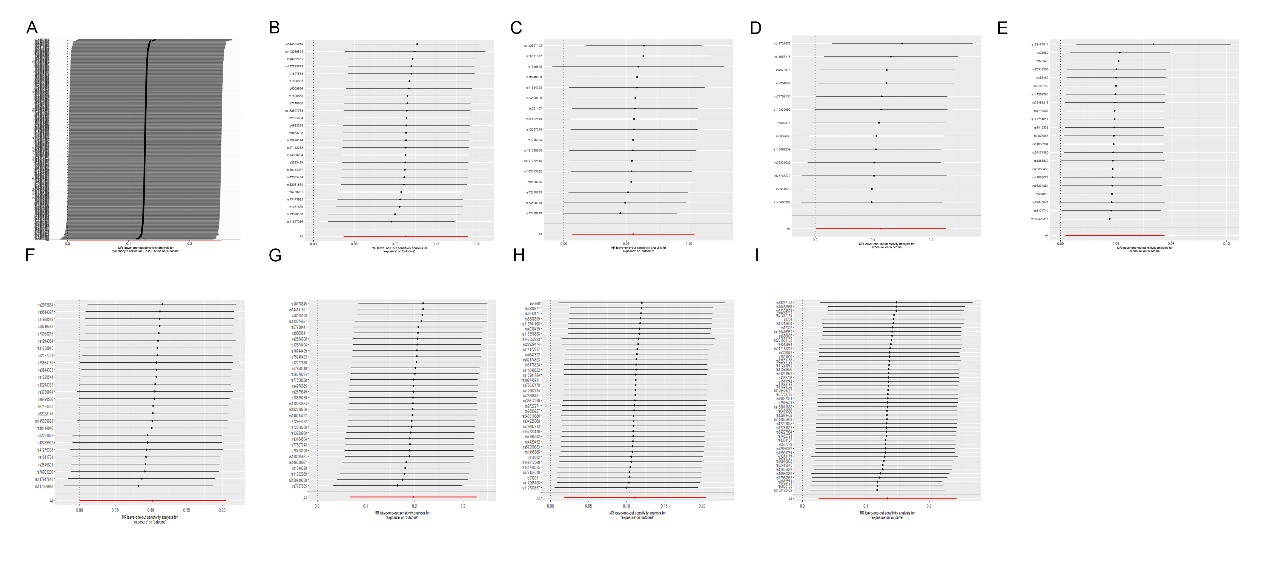
**

**Figure S5 Leave-one-out plots of MR results for nine immune traits.** Leave-one-out plots of MR results for lymphocyte (A), B cell (B), B cell / CD3+ lymphocyte ratio (C), unswitched memory B cell (D), and CD27 on memory B cell (E), TNF (F), TNFR superfamily member 9 (G), TNF-related apoptosis-inducing ligands (H), TNF-related activation-induced cytokines (I). MR, Mendelian randomization; TNF, tumor necrosis factor; TNFL, tumor necrosis factor ligand; TNFR, tumor necrosis factor receptor.

**Figure S6**


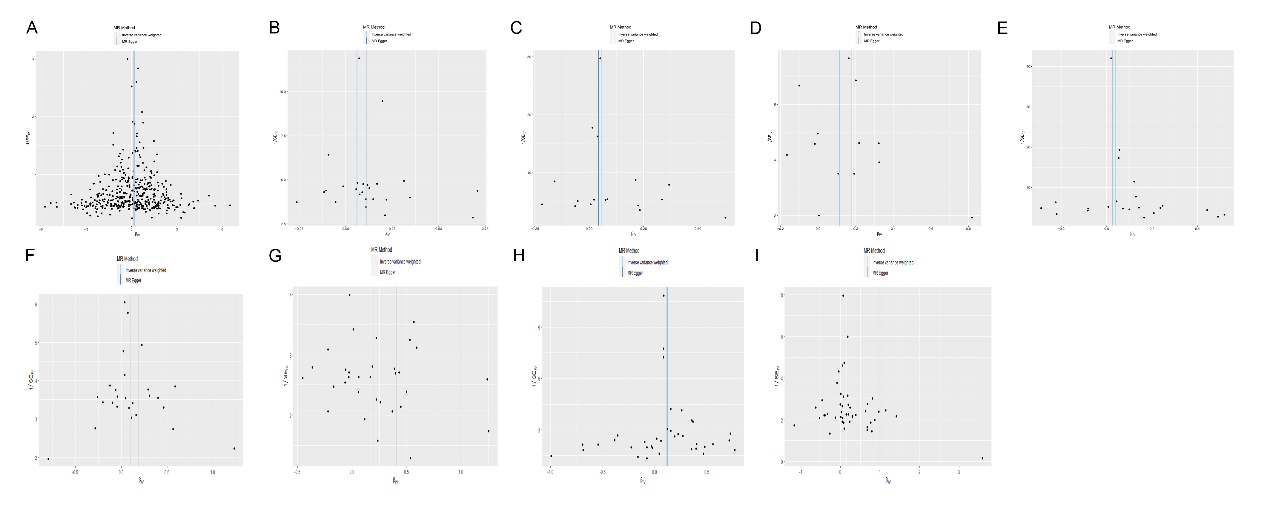


**Figure S6 Funnel plots of MR results for nine immune traits.** Funnel plots of MR results for lymphocyte (A), B cell (B), B cell / CD3+ lymphocyte ratio (C), unswitched memory B cell (D), and CD27 on memory B cell (E), TNF (F), TNFR superfamily member 9 (G), TNF-related apoptosis-inducing ligands (H), TNF-related activation-induced cytokines (I). MR, Mendelian randomization; TNF, tumor necrosis factor; TNFL, tumor necrosis factor ligand; TNFR, tumor necrosis factor receptor.
